# Laparoscopic natural orifice specimen extraction for diverticular disease: a systematic review

**DOI:** 10.1007/s00464-025-11683-8

**Published:** 2025-03-26

**Authors:** Jasmine Mui, Mina Sarofim, Ernest Cheng, Andrew Gilmore

**Affiliations:** 1 Department of Colorectal Surgery, Liverpool Hospital, Liverpool, NSW Australia; 2 St George and Sutherland Clinical School, University of New South Wales, Kogarah, NSW Australia; 3 Faculty of Medicine and Health, University of Sydney, Camperdown, NSW Australia; 4 School of Medicine, Western Sydney University, Liverpool, NSW Australia; 5 Faculty of Medicine, Health and Human Sciences, Macquarie University, Macquarie Park, NSW Australia

**Keywords:** Diverticular disease, Natural specimen orifice extraction, Transrectal extraction, Transanal extraction, Colectomy

## Abstract

**Background:**

Diverticular disease is extremely common in the Western world, with a proportion of complications requiring colonic resection. Whilst laparoscopic surgery has its benefits, a large wound for specimen extraction predisposes to surgical site infection, prolonged pain and prolonged hospital admission. Natural orifice specimen extraction (NOSE) is an alternative technique that has not yet been widely adopted in diverticular disease surgery. The aim of this systematic review is to evaluate the evidence on the outcomes of NOSE in left sided resections for diverticular disease.

**Methods:**

A systematic review of PubMed, Ovid MEDLINE and EMBASE was performed to identify studies that reported outcomes for left sided resections with NOSE in diverticular disease. The studies reviewed were all human studies published in a peer-reviewed journal after 2010. The participants had to be over the age of 18 and the extraction site had to be transanal/transrectal. Articles that were not full text or not in English were excluded. These studies were assessed independently by two reviewers using a standardised pre-piloted form.

**Results:**

One hundred and eighty-seven articles were screened, with 9 articles meeting the inclusion criteria. The study sample size ranged from 8 to 157 participants, with a pooled total of 428 patients who had NOSE. Hospital length of stay varied from 4 to 6 days. Only 2 patients required conversion to transabdominal extraction. Pain scores were lower post-NOSE compared to traditional abdominal extraction in 2 out of 3 studies. The anastomotic leak rate varied from 0 to 18%. Six studies reported no surgical site infections and there was only 1 mortality.

**Conclusion:**

NOSE is a safe and feasible option for patients requiring left sided resection for diverticular disease based on the current available data. The literature demonstrates low rates of surgical site infection, mortality and reduced hospital length of stay.

With the advent of laparoscopic surgery in the 1990s, there has been a shift towards minimally invasive surgery as the mainstay of operative treatment. Laparoscopic surgery has the advantage of reduced pain, reduced hospital stay, improved recovery and improved cosmesis over open operations [1]. However, in traditional laparoscopic colorectal surgery, extraction of the resected specimen requires an extended abdominal incision which can negate the benefits of minimally invasive surgery. These incisions, especially in the elderly or obese population, predispose to wound complications and prolonged admission for pain management [2]. Natural orifice specimen extraction (NOSE) is one technique that allows extraction of a colonic specimen without the need for an extended incision.

There have been a number of randomised controlled trials, systematic reviews and meta-analyses supporting the benefits of NOSE for colorectal cancer [3–5]. However, there is paucity of evidence on the outcomes of laparoscopic NOSE for diverticular disease. Diverticulosis is one of the most common bowel conditions in the western world, affecting 65% of people over the age of 70 [6, 7]. Of those with diverticulosis, 25% will develop diverticulitis and a proportion will have complications, such as abscess, perforation, fistula or stricture [8]. Elective surgery to remove the affected segment of colon has a less than 5% recurrence rate, and is indicated for recurrent attacks of diverticulitis, colovesical fistula and stricture, amongst others [7].

Whilst some institutes have started utilising NOSE for oncological colorectal resections, the adoption of NOSE for diverticular disease has been slow due to concerns that the bulkiness of the inflamed colon and mesentery may inhibit safe transrectal extraction [9]. Furthermore, concerns have been raised regarding the impact of peritoneal contamination during intra-corporeal colonic transection, and this may be further exacerbated by a bulky specimen requiring manipulation for extraction [10–13].

The aim of this systematic review is to examine the recent outcomes of NOSE for diverticular disease, discuss post-operative outcomes and review techniques to manage a bulky specimen.

## Methods

### Search strategy

A targeted systematic review of patients undergoing NOSE for diverticular disease was completed in accordance to Preferred Reporting Items for Systematic Reviews and Meta-Analyses (PRISMA) guidelines [14]. A comprehensive search was conducted in the following electronic databases: PubMed, Ovid MEDLINE and EMBASE. Individual search strategies were tailored to each database using following key terms and Boolean operations (‘AND’, ‘OR’):

1. Natural Orifice Specimen Extraction.
2. Transanal OR Transrectal.
3. Diverticulitis OR Diverticular Disease OR Colon Diverticulum.
4. #1 AND #2 AND #3.
5. #1 AND #2.

### Inclusion and exclusion criteria

To be included, articles had to meet the following criteria:

1. Adult patients (age > 18).
2. Participants undergoing laparoscopic NOSE surgery for diverticular disease.
3. Colon extracted was the left colon only by a transanal or transrectal route.
4. Published in a peer-reviewed journal.

Exclusion criteria were non-human studies, articles that were not in English, articles that did not have a full text, or articles published before January 2010. Non-human studies were excluded due to irrelevance to the population of interest. The reviewers could not accurately or thoroughly assess articles that were not full text or not available in English, and literature published prior to 2010 lacks relevance when considering the rapidly changing landscape of colorectal surgery. Robotic surgery was not included due to the lack of availability of the robotic platform internationally and would thus limit the applicability of this review to current international clinical practice.

Articles that were either opinion pieces or case reports were also not included as these were considered low in the hierarchy of evidence. Systematic reviews and meta-analyses were excluded as these did not contain original full text articles.

### Quality assessment

The methodological quality of the studies was assessed using the modified Newcastle–Ottawa Scale (NOS) for observational studies. Studies with NOS scores of 7 were considered high quality [15].

### Selection of papers

Manuscript assessment was performed independently by two reviewers (JM and MS) using a standardised pre-piloted form. This included study design, baseline characteristics and endpoint outcomes.

### Data extraction and statistical analysis

Data was systematically extracted on age, body mass index (BMI), operative time, conversion to transabdominal extraction (TAE), anastomotic leaks, length of stay (LOS), surgical site infections (SSI), deep infections and mortality. Outcome data was recorded in its reported format of median (range), mean (standard deviation) or absolute percentages.

### Ethics considerations

This study did not require ethics approval as it synthesised published data. It was registered in the PROSPERO database.

## Results

### Study characteristics

**Fig. 1:**
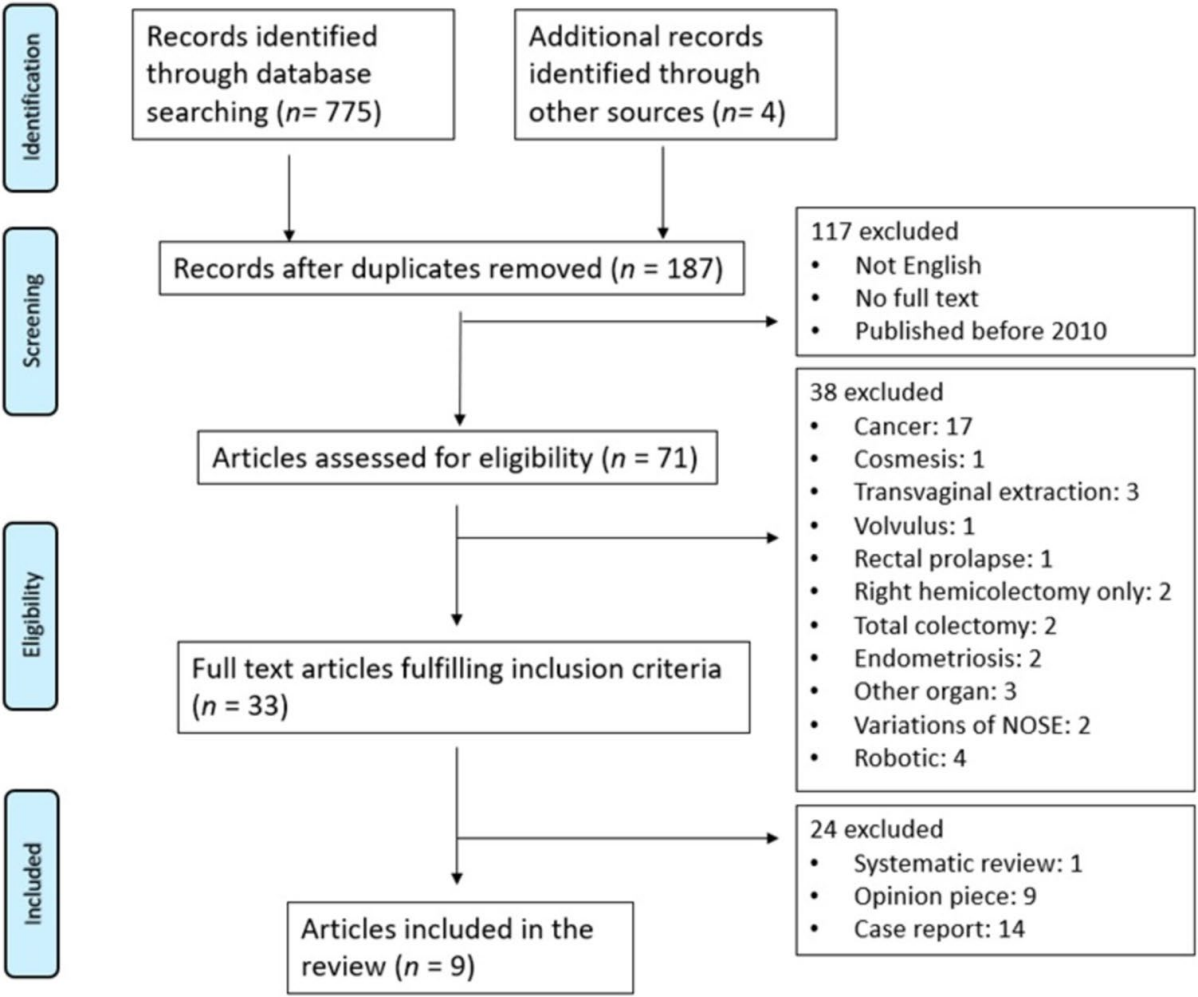
PRISMA flowchart of study selection

After removal of duplicates, a total of 187 articles were retrieved from the database search including 4 additional references found through review of reference lists. A flowchart of the study selection is shown in Fig. 1. Once article abstracts were screened, 117 were excluded because the abstracts were not in English, did not have a full text, or were published before 2010. After full texts were assessed, 38 were excluded as not meeting the eligibility criteria. Of the remaining 33, an additional 24 were excluded. Nine articles were therefore included in the analysis with a pooled total of 563 study participants, 428 of which had NOSE. Of those who had NOSE, at least 119 were for diverticular disease. Characteristics of these studies are listed in Table 1.

**Table 1:** Characteristics of included studies

Author	Year	Study design	Pathology	Study sample size	Number who had NOSE n (%)	Number who had NOSE for diverticular disease n (%)	Age (mean)	BMI (mean)
Saad et al.	2010	Prospective case series	Benign + malignant	8	8 (100)	NA	NA	NA
Wolthuis et al.	2011	Observational case series	Benign + malignant	21	21 (100)	5 (24)	41	23
Costantino et al.	2012	Prospective case matched series	Diverticulitis	38	29 (76)	29 (76)	60	25.5
Leroy et al.	2011	Prospective case series	Diverticulitis	16	16 (100)	16 (100)	61	24.7
Christoforidis et al.	2013	Prospective case series	Benign (diverticulitis, sigmoid volvulus, rectal prolapse)	11	11 (100)	NA	47	27.6
Wolthuis et al.	2015	Randomised controlled trial	Benign + malignant	40	20 (50)	15 (37)	54	23.5
Huang et al.	2017	Retrospective review	Benign + malignant	82	82 (100)	7 (9)	63	24.4
Saurabh et al.	2017	Retrospective review	Benign + malignant	188	82 (44)	NA	63	24.4
Chen et al.	2021	Prospective case series	Benign (diverticulitis, sigmoid volvulus, post-malignant polypectomy)	159	159 (100)	47 (30)	59	28.2

*BMI* Body mass index (kg/m^2^)

### Quality of included studies

There was 1 randomised controlled trial (RCT), 5 prospective studies and the remainder were retrospective in design. Only 2 studies exclusively evaluated diverticular disease; the others reported a heterogenous cohort which included malignant or other benign conditions. Outcomes were not reported separately for each condition. Three studies did not specify the proportion of patients in their study with diverticular disease. Furthermore, there is a high risk of selection bias due to the lack of randomisation of all studies except for the RCT by Wolthuis et al. [16]. Standardised quality assessment was performed using the NOS and outlined in Table 2.

**Table 2:** Quality assessment of the included observational studies using a Modified Newcastle-Ottowa Scale

Study	Selection			Comparability	Outcome		Total
	Representativeness (*)	Selection of non-exposed cohort (*)	Ascertainment of exposure (*)	(**)	Assessment of outcome (*)	Adequacy of follow up (*)	(7*)
Saad et al. [23]	*	-	*	-	*	*	4
Wolthuis et al. [25]	*	-	*	-	*	*	4
Costantino et al. [13]	*	*	*	**	*	*	7
Leroy et al. [11]	*	-	*	-	*	*	4
Christoforidis et al. [9]	*	*	*	*	*	*	6
Wolthuis et al. [15]	*	*	*	**	*	*	7
Huang et al. [21]	*	-	*	-	*	*	4
Saurabh et al. [18]	*	*	*	-	*	*	5
Chen et al. [2]	*	-	*	-	*	*	4

### Patient demographics

The mean age of patients ranged from 41 to 63 years, and mean BMI range was 23–28.2 kg/m^2^. Operating times ranged from 90 to 272 min with only 7 patients requiring conversion from NOSE to TAE.

### Perioperative outcomes

Perioperative outcomes are summarised in Table 3. The highest rate of anastomotic leak was 18% whilst the remaining studies had leak rates ranging from 0 to 5%. Hospital length of stay ranged from 4 to 6 days. 3 patients had a SSI and 4 had a deep abdominal infection. There was only 1 mortality out of the 428 in the pooled NOSE sample.

**Table 3:** Perioperative outcomes of NOSE patients in included studies

Author	Operating time (mins)	Conversion to TAE	Anastomotic leaks n (%)	LOS (days)	SSI n (%)	Deep infection n (%)	Mortality n (%)
Saad et al. [23]	95–180	0	0 (0)	NA	0	0	0
Wolthuis et al. [25]	105	0	1 (5)	6	0	0	1 (5)
Costantino et al. [13]	122	0	1 (3)	6.6	0	1 (3)	0
Leroy et al. [11]	121	0	0 (0)	6.1	0	0	0
Christoforidis et al. [9]	200	1	2 (18)	6	1 (9)	1 (9)	0
Wolthuis et al. [16]	90	1	0 (0)	4	0	0	0
Huang et al. [21]	228	0	2 (2)	5	1 (1)	1 (1)	0
Saurabh et al. [18]	218	0	2 (2)	5.9	0	NA	0
Chen et al. [2]	234	0	5 (3)	5	1 (1)	1 (1)	0

*TAE* Transabdominal extraction, *LOS* length of stay, *SSI* surgical site infection

### Assessment of post-operative pain

Three out of 9 studies compared post-operative pain between patients who had NOSE versus TAE (Table 4). Costantino et al. found that at 24 h, post-operative pain using a visual analogue scale was lower in the NOSE cohort than the TAE cohort, as well as the amount of opiates required [13]. Wolthuis et al. similarly demonstrated post-operative opiate requirements in hospital as well as pain scores one-week post-discharge were both lower in NOSE [16]. However, Christoforidis et al. showed no difference in post-operative pain between the two groups [9].

**Table 4:** Analgesics used during inpatient admission

Wolthuis et al.				Costantino et al		
	NOSE (*n* = 20)	TAE (*n* = 20)	*P* value	NOSE (*n* = 17)	TAE (*n* = 9)	*P* value
Total tramadol requirement (mg)	10.0 (44.7)	40.0 (104.6)	0.298	377.8 (227.9)	671.4 (579.4)	0.02
Total piritramide requirement (mg)	1.1 (4.6)	15.6 (23.8)	0.003	–	–	–
Total paracetamol requirement (g)	11.0 (3.9)	17.0 (1.2)	< 0.001	8.3 (3.3)	15 .1 (9.2)	0.007

Christoforidis et al.			
	NOSE (*n* = 10)	TAE (*n* = 20)	*P* value
Opiate analgesia on last day of admission (mg)	2.5 (0–9)	1 (0–9)	0.160
Total oral morphine equivalent (mg)	72 (18–257)	60 (0–299)	0.214

Values are presented as mean (standard deviation). Values are presented as median (range)

### Faecal incontinence

The studies by Costantino et al. and Wolthuis et al. both discussed post-operative anal continence after NOSE for diverticular disease. Whilst Costantino et al. reported no impairment in anal sphincter function at follow up, it was not specified how this was assessed [13].

Conversely, Wolthuis et al. assessed pre-procedure and post-procedure continence using the Cleveland Clinic Incontinence Score and anal manometry (Basal Pressure and Maximum Squeeze Pressure). There was no significant different in the pre-procedure scores compared to 3 months post-procedure, and no difference between the NOSE and TAE groups [16].

## Discussion

Symptomatic diverticular disease is a common pathology in Western countries. If surgery is required, natural orifice transrectal or transanal extraction is often perceived as not achievable due to the bulkiness of the colon specimen due to mesenteric or bowel wall inflammation, or theoretical damage the anal sphincter. This is one of the reasons NOSE is more likely to be performed for small colonic malignancies than diverticular disease [9]. NOSE colectomy has been shown, in meta-analyses and RCTs, to reduce hospital length of stay, opiate requirements and infection rates over traditional TAE in left sided resections for colorectal cancer [3–5]. This is the first systematic review of the literature to evaluate the outcomes of NOSE specifically for diverticular disease, which highlights the safety profile and achievable benefits contrary to postulated concerns. This review focused on the transanal or transrectal approach rather than the much less common transvaginal route due to the separate risk profile of adverse events. Completion of NOSE without the need to convert to TAE was 99.5% % based on the pooled data of the included studies.

Not surprisingly, decreased LOS reduces the burden on the healthcare system and also decreases patient risk of venous thromboembolism and hospital acquired infections. Amongst the studies in our review, LOS ranged from 4 to 6 days which is shorter than the mean 5–9 days in the published literature for TAE [17]. In the study by Saurabh et al., direct comparison between LOS in the NOSE cohort with the TAE cohort demonstrated an overall shorter LOS by one day (4.8 vs 5.9, respectively) [18].

Post-operative pain is typically managed by a multimodal approach which includes oral medications, percutaneous catheters and/or intravenous patient-controlled analgesia (PCA). Opiates increase the incidence of post-operative ileus by their unintended inhibition of intestinal acetylcholine receptors, and intravenous PCA may restrict patient mobility. In the study by Wolthuis et al. NOSE patients required lower doses of the opioids compared to their TAE counterparts [16]. Furthermore, pain scores were significantly lower one-week post-discharge, which has been reflected in other studies in the literature [10, 12, 13]. Reduction in post-operative pain has been shown to reduce long term adverse outcomes by reducing the neurohumoral stress response and improving normalisation of bowel function [16]. NOSE is thus a worthwhile alternative for patients who require surgery for diverticular disease particularly if they are at risk of deconditioning or bowel dysregulation.

One trade-off in undertaking NOSE is the longer operating time compared to laparoscopic TAE procedures, which has been shown to range from 142 to 180 min in multicentre trials [17]. Six of the 9 studies in this review reported a mean operative time of 2 h or less, with the variability in operating times likely reflecting the differing volumes of NOSE performed at each institute. With the mastery of the learning curve, operative times would be expected to improve, similar to trends seen during the early days of laparoscopic surgery [1]. Christofordis et al. found that operative times were actually similar between their NOSE and TAE cohorts, and felt that the additional time spent on the NOSE technique was equal to the time it spent to close the lower midline laparotomy incision in TAE [9]. Although Chen et al.s paper reported the longest mean operating time, their cohort of patients had the highest average BMI and encompassed a greater proportion of complex diverticular pathology [2].

Surgical site infection (SSI) was scarce in the studies reviewed, with only 3 of the 428 patients (1%) having a superficial surgical site infection. This is much lower than the rate of SSI in laparoscopic TAE colorectal surgery [19]. Four patients (1%) had a deep pelvic collection managed with either antibiotics or IR drainage. In studies specifically reviewing peritoneal fluid washings, both Costantino et al. and Leroy et al. found that mixed bacterial growth was present on all samples taken intra-operatively, however only 1 out of 29 of patients in Costantino et al.’s study and none in Leroy et al.’s study developed a deep pelvic infection [11, 13]. Furthermore, there was no difference in deep infection rates when compared to the matched TAE group in Costantino et al.’s study, which is similar to results seen in other matched NOSE studies [10, 12, 13]. Given that there is also a degree of contamination during TAE, it is unlikely that the degree of peritoneal contamination during NOSE is any more significant, and the presence of peritoneal bacteria does not correlate to increased rates of deep infection [10, 13].

Anastomotic leak rate is an important key performance indicator that is directly related to patient morbidity. Rates of 5–19% are reported internationally in colorectal anastomoses [20]. In this review, the highest anastomotic leak rate was 18%, which those authors theorised was due to a technical failure of the intra-corporeal anastomosis in the 2 patients who had a leak [9]. They changed their technique from an end-to-end anastomosis to a side-to-end anastomosis which subsequently did not demonstrate any anastomotic compromise. Conversely, Saurabh et al. found no difference between leak rate and pelvic abscess rate in their NOSE patients compared to their matched TAE patients [18]. The variability in the way the intra-corporeal anastomosis is performed between studies highlights the technical proficiency and advanced laparoscopic skills required for successful NOSE [21]. It is for this reason some surgeons will consider NOSE on robotic platforms for the added instrument manoeuvrability and visualisation [22].

In Chen et al.’s study, 30% of their patients had NOSE for diverticular disease and 48% of specimens required de-fatting of the mesentery to allow transrectal extraction [2]. A subset of those patients required further de-bulking by dividing the colonic specimen longitudinally. De-fatting of the mesentery has also been described in the study by Saad et al., and in a case report by Yagci et al. who found success in delivering a colonic specimen with an initial diameter of 12cm [23, 24]. Furthermore, in Chen et al.’s study, abscess formation and SSI was not increased in those patients who required longitudinal colonic division to facilitate extraction [2]. This may be attributed to pre-operative bowel preparation and intra-operative colonic povidone-iodine irrigation that enables safer transection and manipulation of colonic specimens without gross peritoneal soilage. De-bulking manoeuvres understandably further increases operative time, with the average operating time for Chen et al. being the highest of the studies reviewed, but this may also be due to their cohort having the highest BMI.

Continence does not appear to differ significantly between NOSE and TAE patients. Costantino et al. theorised that this was due to the good compliance of the rectum and anus in allowing the extraction of bulky specimens without resistance [13]. This is supported by Wolthuis et al. and Gundogan et al. who found no difference between incontinence scores or anal manometry of patients who had NOSE compared to TAE [12, 16].

This systematic review is limited by the reliance on a small number of heterogenous studies that exist regarding laparoscopic NOSE colectomy for diverticular disease. Apart from the studies by Costatino et al. and Leroy et al., the other studies in this systematic review had a combination of benign and/or malignant conditions and thus the outcomes reported are not specific to diverticular disease. There were, however, a large proportion of patients with diverticular disease that made up the cohorts of at least 3 of the studies. There is a need for a more robust prospective cohort of patients, without heterogeneity of other colonic diseases or cancer in the reporting of outcome data. The only randomised trial was published in 2015 which reflects the relative infancy of this technique. This was the only study whose participants were randomised; thus, the remaining studies pose significant selection bias when interpreting the results. Some studies were also done at institutes that were at varying stages in the NOSE learning curve and this may explain variability in operating times and complication rates.

There were two studies by Wolthius et al. included in this review [16]. The first study in 2011 was an observational study of consecutive patients from July 2009 to February 2010 who underwent NOSE, whereas the second study was a prospective RCT of patients recruited from December 2009 to September 2013. Theoretically there may be a redundancy in patients from December 2009 to February 2010, but this is likely to equate to only a small number of duplicate patients given this is only a 3-month period of overlap.

## Conclusion

This is the first systematic review of laparoscopic colectomy with NOSE for diverticular disease. It is an infrequently performed technique but should be considered for patients with high risk of wound complications or poor tolerance to pain. It appears safe and offers patients lower post-operative pain, reduced risk of surgical site infections and shorter length of stay without an increased risk of deep abdominal infection or incontinence. The limited literature also shows that a bulky specimen or high BMI do not completely preclude a patient from being eligible for NOSE as there are intra-operative strategies for de-bulking. The review is limited by the lack of homogenous data on diverticular disease, as a lot of the data is mixed with other benign or malignant conditions.
